# Relationship between sleep duration and TV time with cardiometabolic risk in adolescents

**DOI:** 10.1186/s12199-020-00880-7

**Published:** 2020-08-21

**Authors:** Ana Paula Sehn, Anelise Reis Gaya, Arieli Fernandes Dias, Caroline Brand, Jorge Mota, Karin Allor Pfeiffer, Javier Brazo Sayavera, Jane Dagmar Pollo Renner, Cézane Priscila Reuter

**Affiliations:** 1 Graduate Program in Health Promotion, University of Santa Cruz do Sul (UNISC), Santa Cruz do Sul, RS Brazil; 2 Graduate Program in Human Movement Sciences, Physical Education, Physiotherapy and Dance School, Federal University of Rio Grande do Sul (UFRGS), Porto Alegre, RS Brazil; 3 Research Center on Physical Activity, Health and Leisure (CIAFEL), Faculty of Sport, University of Porto, Porto, Portugal; 4 Department of Kinesiology, Michigan State University, East Lansing, USA; 5 Centro Universitario Regional Noreste, Universidad de la República, Rivera, Uruguay; 6 Graduate Program in Health Promotion, Department of Life Sciences, University of Santa Cruz do Sul (UNISC), Santa Cruz do Sul, RS Brazil; 7 Graduate Program in Health Promotion, Department of Health Sciences, University of Santa Cruz do Sul (UNISC), Av. Independência, 2293, Santa Cruz do Sul, RS 96815-900 Brazil

**Keywords:** Sleep; Television; Metabolic syndrome; Adolescent

## Abstract

**Objective:**

To verify the association between sleep duration and television time with cardiometabolic risk and the moderating role of age, gender, and skin color/ethnicity in this relationship among adolescents.

**Methods:**

Cross-sectional study with 1411 adolescents (800 girls) aged 10 to 17 years. Television time, sleep duration, age, gender, and skin color/ethnicity were obtained by self-reported questionnaire. Cardiometabolic risk was evaluated using the continuous metabolic risk score, by the sum of the standard *z*-score values for each risk factor: high-density lipoprotein cholesterol, triglycerides, glycemia, cardiorespiratory fitness, systolic blood pressure, and waist circumference. Generalized linear regression models were used.

**Results:**

There was an association between television time and cardiometabolic risk (*β*, 0.002; 95% CI, 0.001; 0.003). Short sleep duration (*β*, 0.422; 95% CI, 0.012; 0.833) was positively associated with cardiometabolic risk. Additionally, age moderated the relationship between television time and cardiometabolic risk (*β*, − 0.009; 95% CI, − 0.002; − 0.001), suggesting that this relationship was stronger at ages 11 and 13 years (*β*, 0.004; 95% CI, 0.001; 0.006) compared to 13 to 15 years (*β*, 0.002; 95% CI, 0.001; 0.004). No association was found in older adolescents (*β*, 0.001; 95% CI, − 0.002; 0.002).

**Conclusions:**

Television time and sleep duration are associated with cardiometabolic risk; adolescents with short sleep have higher cardiometabolic risk. In addition, age plays a moderating role in the relationship between TV time and cardiometabolic risk, indicating that in younger adolescents the relationship is stronger compared to older ones.

## Introduction

In recent years, there has been an increase in cases of Brazilian adolescents with cardiometabolic risk [1]. This situation has raised concerns among health professionals, as it is related to the emergence of cardiovascular diseases [2] and other health disorders [3]. Several lifestyle factors are known to influence the early development of cardiometabolic risk and the occurrence of cardiovascular diseases, such as time spent watching television (TV) [4], sleep duration [5], physical inactivity, and food habits [6]. In addition, excess body weight is also a factor that seems to be related to the development of these diseases [7, 8].

In adolescence, sleep is associated with the physical and mental development [9]. Nevertheless, due to the various activities performed during the day, adolescents sleep fewer hours than recommended [9] and this can reflect on quality of life, influencing the early onset of cardiovascular disease. Indeed, recommendations for bedtime appear to be disregarded since childhood, and the early risk of developing mental, cardiometabolic, musculoskeletal disorders also seems to be associated with this behavior [10]. However, there is still little evidence in the literature about the deleterious effects of sleep duration on adolescent cardiometabolic diseases [11] and the possible moderating variables of this relationship.

TV and screen time have also been associated with adolescents’ cardiometabolic health [12]. According to the American Academy of Pediatrics, adolescents should spend a maximum of 2 h a day in front of screens [13]. This recommendation is due to the fact that high sedentary behavior is associated with the early risk of a series of chronic diseases, and such behavior still seems to be independent of regular physical activity practice [14, 15]. Although evidence is clear in the adult population, there is still limited information in the youth population [15].

In addition, factors such as age, gender, and skin color/ethnicity have also shown evidence of moderating effects in relation to cardiometabolic risk. In the study conducted by Vliet et al. [16], which compared individuals from different countries, there were differences between ethnicities and gender in the development of cardiometabolic risk [17]. The same occurred with age, which showed a difference between adolescents and pre-adolescents [18]. In this sense, unlike the relationship between obesity and cardiometabolic diseases that is already established in the literature, there is limited evidence of the independent relationship between TV time and sleep duration with cardiometabolic risk, especially in Brazilian adolescents. Thus, this study aimed to verify the association between sleep duration and TV time with cardiometabolic risk and the moderating role of age, gender, and color/ethnicity in this relationship in adolescents.

## Methods

This cross-sectional study was conducted with 1411 adolescents aged 10 to 17 years, of both genders (800 females), who were from public and private schools and were residents of the urban and rural area (north, south, east, west, and center) of a municipality in southern Brazil. This study was conducted in 2016 and 2017 and approved by the Research Ethics Committee of the University of Santa Cruz do Sul (UNISC) under Opinion No. 2.936.223. All methods were performed in accordance with the relevant guidelines and regulations, and informed consent was obtained from all participants.

**Fig. 1:**
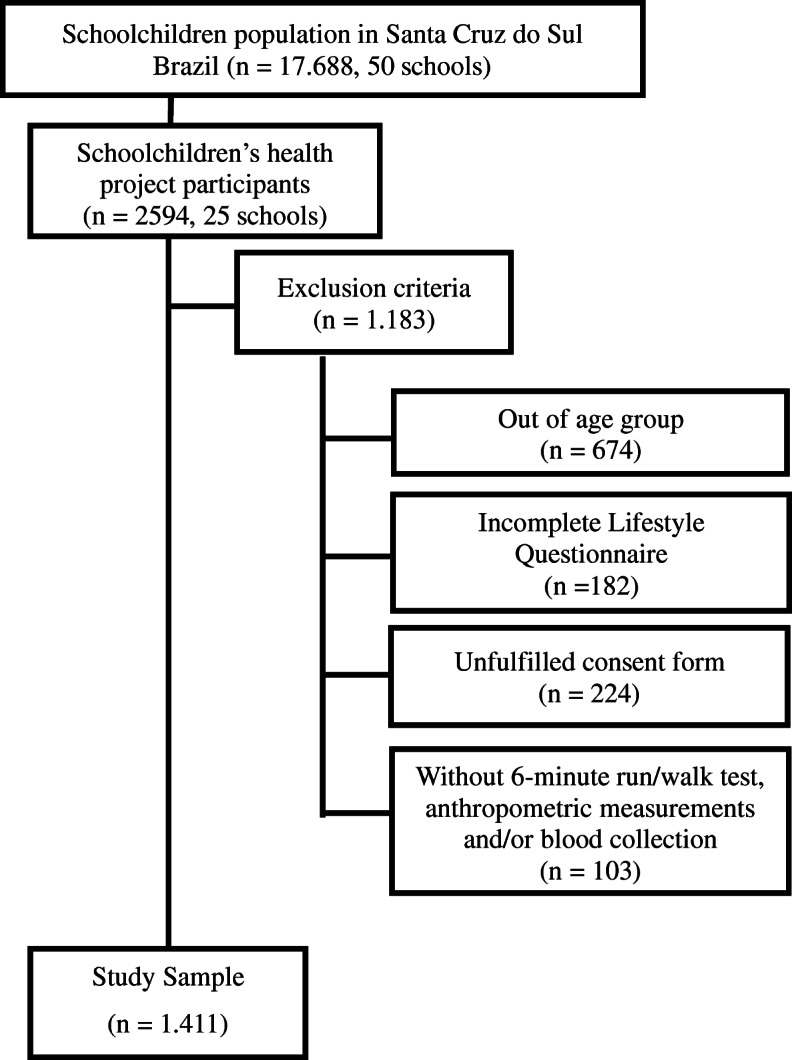
Sample selection flowchart

Since 2004, the same schools have been invited to participate in the research, in order to form a cohort. A survey was conducted in the city of Santa Cruz do Sul, Rio Grande do Sul State, which indicated the number of schools (*n* = 50) and schoolchildren (*n* = 17,688) enrolled. From this, a sample size calculation was made considering the population density of schoolchildren in all regions of the municipality, including public (municipal and state) and private networks. Afterwards, schools were randomly selected. To select the subjects of the present study, the following inclusion criteria were determined: ages between 10 and 17 years and authorization by parents or guardians by signing the informed consent form, as well as the consent form for students aged 12 years and over, and as exclusion criteria: do not fill in the lifestyle questionnaire correctly and do not perform blood collection and the cardiorespiratory fitness (CRF) test. Data collection was developed at the facilities of the university (Fig. 1).

Sample size calculation was performed using the G*Power 3.1 program (Heinrich-Heine-Universität—Düsseldorf, Germany). Multiple linear regression was considered by Faul et al. [19] the most appropriate statistical test to be applied, estimating a minimum sample of 995 students, using the following reference parameters: test power (1 − *β*) = 0.95, effect size (*f*²) = 0.02, and significance level *α* = 0.05.

### Collection instruments

To evaluate sleep duration, TV time, and information regarding color/ethnicity and age, the adolescents answered a questionnaire. Sleep pattern was assessed by the following questions: “what time do you usually sleep during the week?/weekend? ”and “what time do you usually wake up during the week?/weekend?” After obtaining the week and weekend sleep times, both were averaged to determine the total sleep duration of the week. From this, sleep was classified according to the reference values of the National Sleep Foundation [20]. For individuals aged 10 to 13 years, it was considered ≤ 8 h as short time, 9 to 11 h for adequate sleep time, and ≥ 12 h for long sleep. For adolescents aged 14 to 17 years, it was classified as ≤ 7 h (short time), 8 to 10 h (adequate time), and ≥ 11 h (long time). TV time was assessed by asking “indicate the number of hours or minutes you watch TV per day.” The response was transformed into minutes.

### Cardiometabolic risk assessment

Blood collection from the brachial vein was performed by a trained professional in an appropriate room. Serum samples were used for biochemical analyses of the following parameters: high-density lipoprotein cholesterol (HDL-c), triglycerides (TG), and glycemia, after 12 h of fasting. Analyses were performed on the Miura 200 automated device (I.S.E., Rome, Italy) using serum samples and commercial Kovalent/DiaSys kits (DiaSys Diagnostic Systems, Germany). For HDL-c and TG the reference values of the National Heart, Lung, and Blood Institute [21] were used*.* Glycemia was classified according to the protocols of the American Diabetes Association [22]*.*

To assess CRF, the 6-min run/walk test was used, performed on the athletic track, using the distance covered in meters [23]. Then, the peak oxygen uptake (VO_2peak_) formula was applied = 41.946 + 0.022(6 min) − 0.875 (body mass index) + 2.107 (gender, considering 1 for boys and 0 for girls) to transform the distance into meters for VO_2peak_ [24].

Blood pressure was measured by auscultation using a sphygmomanometer, stethoscope on the right arm, and a cuff appropriate to the individual’s arm circumference. For this, it was indicated that the adolescents remained seated and resting for 5 min. Two measurements were made, using the lowest value of systolic blood pressure (SBP). Waist circumference (WC) was evaluated using the narrowest part of the trunk between ribs and iliac crest, using an inelastic tape with a resolution of 1 mm (Cardiomed®).

Cardiometabolic risk was evaluated using the continuous metabolic risk score (cMetS), by the sum of the standard *z*-score values for each risk factor: HDL-c, TG, glycemia, SBP, WC, and CRF [25]. To calculate the *z*-score, the following form was used: *z*-score ([value of continuous variable − cutoff points]/standard deviation). The cutoff points and standard deviation used were based on the study of Stavnsbo et al. [26]. Considering that CRF and HDL-c present an inverse relationship with cardiometabolic risk, the score of these variables was multiplied by − 1. Age and gender were used to calculate the *z*-score of the variables individually [25]. Also, the inclusion of CRF in the cardiometabolic score have been suggested in the literature [27].

### Statistical analysis

Statistical analyses were performed using the Statistical Package for the Social Sciences (SPSS) v. 23.0 (IBM, Armonk, USA). To describe the sample, descriptive statistics were used through absolute and relative frequency, as well as mean and standard deviation. Chi-square and Student’s *t* tests were used to compare groups according to gender. Generalized linear models were tested: model 1, association between TV time, age, gender, and skin color/ethnicity with cardiometabolic risk; model 2, association between TV time, age, and interaction (TV time × age) with cardiometabolic risk; model 3, association between TV time, gender, and interaction (TV time × gender) with cardiometabolic risk; and model 4, association between TV time, skin color/ethnicity, and interaction (TV time × skin color/ethnicity) with cardiometabolic risk. The same models were used for sleep duration, in which TV time is replaced by sleep duration. The variables that showed interaction were tested in the PROCESS macro for the SPSS program using linear regression models.

## Results

The sample characteristics are presented in Table 1. The results indicated that 23.2% have short sleep duration. Regarding the means, boys presented higher VO_2peak_ and lower TV time.

**Table 1:** Descriptive characteristics of adolescents according to gender

	***n*** (%)			***p***
	Total (***n*** = 1411)	Male (***n*** = 611)	Female (***n*** = 800)	
**Skin color/ethnicity**				
White	1121 (79.4)	482 (78.9)	639 (79.9)	0.879
Black	97 (6.9)	45 (7.4)	52 (6.5)	
Brown/mulatto	174 (12.3)	74 (12.1)	100 (12.5)	
Indigenous	10 (0.7)	5 (0.8)	5 (0.6)	
Yellow	9 (0.6)	5 (0.8)	4 (0.5)	
**Sleep duration**				
Short time	328 (23.2)	157 (25.7)	171 (21.4)	0.152
Adequate	1015 (71.9)	427 (69.9)	588 (73.5)	
Long time	68 (4.8)	27 (4.4)	41 (5.1)	
	**Mean (SD)**			***p***
Age (years)	12.74 (1.96)	12.87 (1.99)	12.65 (1.93)	0.416
TV time (min)	142.50 (120.85)	132.69 (110.21)	149.39 (127.42)	**0.008**
CR (*z*-score)	− 0.23 (3.31)	− 0.29 (3.19)	− 0.18 (3.40)	0.235
WC (cm)	67.99 (8.93)	69.68 (9.17)	66.69 (8.52)	0.146
VO_2peak_ ((mL/kg)/min)	44.41 (6.73)	48.09 (6.49)	41.60 (5.44)	**< 0.001**
SBP (mmHg)	107.57 (12.97)	108.29 (12.72)	107.02 (13.14)	0.945
TG (mg/dL)	71.46 (31.62)	66.09 (30.07)	75.56 (32.17)	0.053
HDL-c (mg/dL)	57.17 (10.47)	57.05 (10.90)	57.26 (10.12)	0.052
Glycemia (mg/dL)	88.50 (6.89)	90.00 (6.78)	87.35 (6.76)	0.395

*SD* standard deviation, *CR* cardiometabolic risk, *WC* waist circumference, *VO*_*2peak*_ peak oxygen uptake, *SBP* systolic blood pressure, *TG* triglycerides, *HDL-c* high density lipoprotein cholesterol; chi-square test and Student *t* test; *p* < 0.05

The associations between sleep duration, age, gender, and skin color/ethnicity with cardiometabolic risk are shown in Table 2. In models 1 and 4, a positive association was found among adolescents who reported short sleep duration (*β*, 0.422; CI 95 %, 0.012; 0.833; *β*, 0.525; 95% CI, 0.062; 0.988) with cardiometabolic risk, in relation to those with adequate sleep duration. However, no significant interaction of sleep duration with age, gender, and skin color/ethnicity was found.

**Table 2:** Regression analysis between sleep duration, age, gender, and skin color/ethnicity with cardiometabolic risk in adolescents

	***β***	Cardiometabolic risk
		CI (95%)
**Model 1 (AIC, 7381.388; BIC, 7433.909)**		
**Sleep duration**		
Adequate	1	
Short time	**0.422**	(0.012; 0.833)
Long time	0.906	(0.098; 1.715)
Age	− 0.059	(− 0.147; 0.029)
**Gender**		
Male	1	
Female	0.102	(− 0.245; 0.449)
**Skin color/ethnicity**		
White	1	
Black	**− 0.990**	(− 1.673; − 0.307)
Brown/mulatto	− 0.419	(− 0.945; 0.107)
Indigenous	1.067	(− 0.982; 3.116)
Yellow	− 1.029	(− 3.186; 1.128)
**Model 2 (AIC, 7383.571; BIC, 7446.596)**		
**Sleep duration**		
Adequate	1	
Short time	2.283	(− 0.486; 5.053)
Long time	0.729	(− 4.842; 6.300)
Age	− 0.028	(− 0.130; 0.075)
**Sleep duration × age**		
Adequate × age	1	
Short time × age	− 0.145	(− 0.357; 0.068)
Long time × age	0.014	(− 0.422; 0.450)
**Model 3 (AIC, 7383.749; BIC, 7446.773)**		
**Sleep duration**		
Adequate	1	
Short time	0.293	(− 0.309; 0.895)
Long time	0.292	(− 0.988; 1.573)
**Gender**		
Male	1	
Female	− 0.002	(− 0.412; 0.408)
**Sleep duration × gender**		
Adequate × gender (ref. male)	1	
Short time × gender	0.236	(− 0.586; 1.058)
Long time × gender	1.021	(− 0.630; 2.671)
**Model 4 (AIC, 7392.218; BIC, 7486.755)**		
**Sleep duration**		
Adequate	1	
Short time	**0.525**	(0.062; 0.988)
Long time	**1.103**	(0.139; 2.068)
**Skin color/ethnicity**		
White	1	
Black	**− 0.861**	(− 1.708; − 0.014)
Brown/mulatto	− 0.300	(− 0.932; 0.331)
Indigenous	1.823	(− 0.621; 4.267)
Yellow	− 0.281	(− 2.915; 2.354)
**Sleep duration × Skin color/ethnicity**		
Adequate × white	1	
Short time × black	− 0.260	(− 1.813;1.293)
Short time × brown/mulatto	− 0.514	(− 1.739; 0.712)
Short time × indigenous	− 2.339	(− 7.522; 2.844)
Short time × yellow	− 0.401	(− 5.674; 4.872)
Long time × black	− 0.845	(− 3.447; 1.757)
Long time × brown/mulatto	0.064	(− 2.179; 2.308)
Long time × indigenous	− 2.982	(− 9.927; 3.963)
Long time × yellow	− 6.057	(− 13.070; 0.956)

*CI* confidence interval, *AIC* Akaike information criterion, *BIC* Bayesian information criterion, *β* linear regression

Table 3 shows the association between TV time, age, gender, and skin color/ethnicity with cardiometabolic risk. In model 1, a weak positive association between TV time and cardiometabolic risk was found (*β*, 0.002; 95% CI, 0.001; 0.003). For example, if the adolescent remains 10 min in front of the TV, it corresponds to an increase of 0.02 in the cardiometabolic risk score. However, model 2 indicates a negative interaction between TV time × age with cardiometabolic risk (*β*, − 0.001; 95% CI, − 0.002; − 0.001), demonstrating that as age increases, the relationship between TV time and cardiometabolic risk decreases.

**Table 3:** Regression analysis between TV time, age, gender, and skin color/ethnicity with cardiometabolic risk in adolescents

	***β***	Cardiometabolic risk
		CI (95%)
**Model 1 (AIC, 6062.282; BIC, 6107.834)**		
TV time	**0.002**	(0.001; 0.003)
Age	− 0.029	(− 0.125; 0.066)
**Gender**		
Male	1	
Female	0.010	(− 0.369; 0.388)
**Skin color/ethnicity**		
White	1	
Black	− 1.252	(− 1.998; − 0.540)
Brown/mulatto	− 0.571	(− 1.139; − 0.003)
Indigenous	1.044	(− 1.214; 3.302)
Yellow	− 1.252	(− 3.506; 1.002)
**Model 2 (AIC, 6058.391; BIC, 6108.814)**		
TV time	0.015	(0.005; 0.026)
Age	0.109	(− 0.036; 0.255)
TV time × age	**− 0.001**	(− 0.002; − 0.001)
**Model 3 (AIC, 6064.084; BIC, 6114.698)**		
TV time	0.002	(0.001; 0.004)
**Gender**		
Male	1	
Female	0.113	(− 0.477; 0.702)
TV time × gender (ref. male)	1	
TV time × gender	− 0.001	(− 0.004; 0.003)
**Model 4 (AIC, 6068.321; BIC, 6134.118)**		
TV time	0.009	(− 0.011; 0.029)
**Skin color/ethnicity**		
White	1	
Black	− 1.396	(− 2.497; − 0.296)
Brown/mulatto	− 0.687	(− 1.558; 0.184)
Indigenous	2.541	(− 0.905; 5.986)
Yellow	− 2.783	(− 7.683; 2.116)
TV time × white	1	
TV time × black	0.001	(− 0.004; 0.006)
TV time × brown/mulatto	0.001	(− 0.003; 0.005)
TV time × indigenous	− 0.006	(− 0.017; 0.005)
TV time × yellow	0.007	(− 0.013; 0.027)

*CI* confidence interval, *AIC* Akaike information criterion, *BIC* Bayesian information criterion, *TV* television, *β* linear regression

**Fig. 2:**
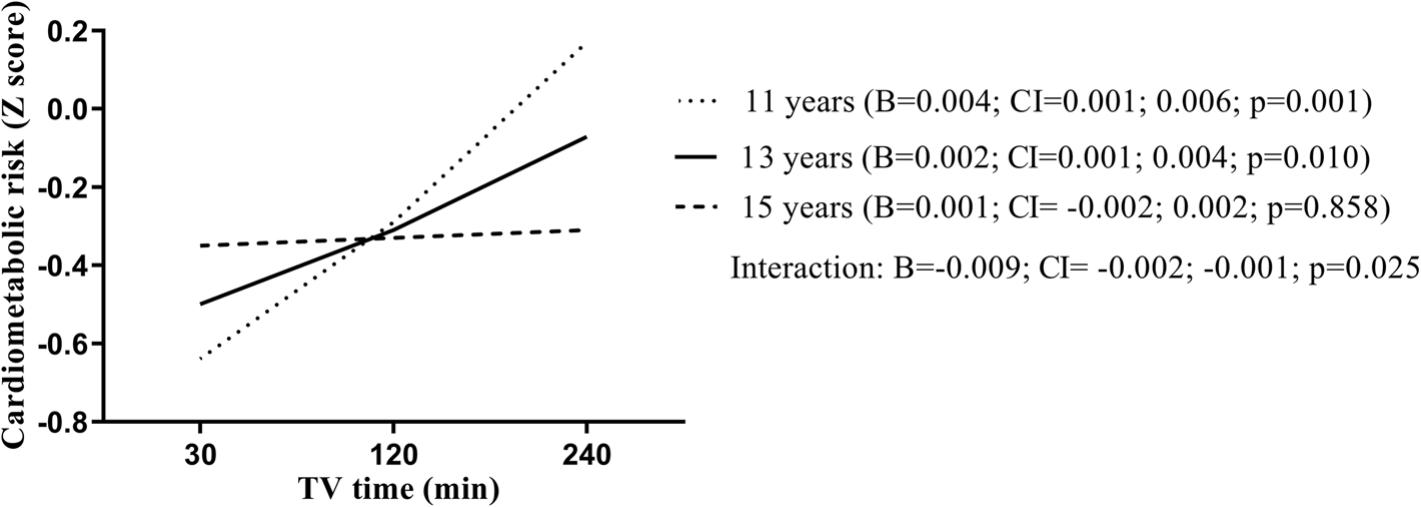
Age as a moderator in the relationship between TV time and cardiometabolic risk. TV, television; *β*, linear regression

Age was a moderator of the relationship between TV time and cardiometabolic risk (Fig. 2). There is a significant relationship between TV time and cardiometabolic risk, and this association is stronger in younger adolescents. However, no relationship was found for adolescents aged 15 years or older.

## Discussion

Our study showed a positive association between short sleep with cardiometabolic risk in Brazilian adolescents. TV time was also moderated by age with cardiometabolic risk, suggesting that in younger adolescents this association was stronger. Therefore, younger adolescents who do not comply with TV time recommendations have higher cardiometabolic risk values compared to older adolescents.

The fact that sleep is related to cardiometabolic risk deserves attention and corroborates findings in the literature [5, 10], especially in adolescents with short sleep [28, 29]. In adults, similar behavior is observed, indicating that the ones with short sleep duration are more likely to develop cardiovascular disease than individuals with adequate sleep duration [28]. The association with short sleep duration can be explained by changes in adolescents’ lifestyle, such as greater exposure to screens, participation in parties, new affective and social relationships, more developed activities, going out to parties [9], the shift that the student studies at school, preferably in the morning and night shifts, and a longer time for sedentary behavior [30]. These changes alter sleep quality and increase the chances of adolescents developing cardiometabolic impairments [29]. However, it is important to note that insufficient or prolonged sleep becomes a risk to cardiometabolic health in adolescents, mainly because there is already evidence in the literature, indicating that the short and long duration of sleep increase the risk of mortality from various causes [31, 32].

Therefore, it seems clear that sleep may be related to the development of these cardiometabolic changes in young people. However, in Brazil, there are still few studies, and international evidence is mainly derived from cross-sectional studies [11]. This evidence suggests the need for health programs that emphasize the importance of maintaining adequate sleep quality and quantity, as well as the encouragement of parents to adopt a healthy lifestyle [29], in order to reduce cardiometabolic diseases resulting from these changes in sleep and to maintain a better quality of life [29, 33].

Regarding TV time, it was found that this variable is an important behavior to be considered for cardiometabolic risk of adolescents [34], whereas in order to maintain an adequate cardiometabolic risk profile, it is important to reduce the time in front of the screens or limit this exposure to 2 h [13]. However, our study suggests that TV time seems to have a stronger association in the early development of cardiometabolic risk in younger adolescents compared to older ones. We suggest that this evidence deserves attention from a public health perspective. Younger adolescents appear to be more harmed by time in sedentary behavior compared to their older peers. One hypothesis for this finding is that younger adolescents spend more time in front of the TV [35] and in other sedentary behaviors and present lower levels of physical activity and higher body adiposity [36], than older adolescents. Also, urbanization caused a reduction in the safety of public places intended for adolescents to engage in physical activities [37].

Moreover, our results corroborate those found by Carson and Janssen [38], in which it was observed that individuals with high TV time were more likely to present altered cardiometabolic risk profile than individuals with low TV time. Another study of 11- and 13-year-old obese adolescents also found that reducing screen time is beneficial for cardiometabolic health [35]. Also, lower cardiometabolic risk values were observed in adolescents who comply with the recommended levels of physical activity and low sedentary behavior [39]. However, it is noteworthy that the evidence on the relationship between TV time and cardiometabolic risk is still inconsistent [40, 41]. Many authors have shown that TV time becomes risky behavior when children are inactive [40, 42], while others suggest an increased health risk when recommendations are not met, regardless of other behaviors [14, 43]. In this sense, it is also observed that high exposure to screens is associated with greater intake of unhealthy foods and excess body weight, behaviors that increase the chances of developing cardiometabolic risk factors [44].

Given the high prevalence of sedentary behavior, especially time in front of TV in Brazilian adolescents [45], and its association with the presence of cardiometabolic risk, it is important to carry out health actions, such as lifestyle interventions, in order to reduce the time spent on screens [35] in an attempt to replace it with physical activity [12] and, consequently, minimize the effects that this behavior has on the cardiometabolic health of these individuals [12, 45–47]. For overweight and obese adolescents, it is suggested to reduce screen time and increase physical activity levels, mainly because obesity is already associated with the development of cardiovascular diseases [48]. However, for normal weight individuals, just raising physical activity levels would be enough to reduce cardiometabolic diseases [49]. Therefore, it is suggested that reducing cardiometabolic risk and preventing chronic diseases in adolescents, regardless of age, it is essential to decrease screen and TV time and increase physical activity levels [15, 47, 50].

### Limitation and strong point

Some limitations need to be pointed out, such as the fact that TV and sleep duration are self-reported by adolescents through a questionnaire, which may not be in line with reality; we do not consider the use of other screens, such as smartphones and tablets, for example, and the exposure in front of the cell phone has not been evaluated. In addition, other behaviors that may interfere in the investigated relationship, such as physical activity, eating habits, and nutritional status were not considered. Also, motivational, educational, and family factors were not approached as potential confounders. However, the inclusion of a representative sample of a southern Brazilian city becomes a strong point of the study, especially since there are few studies with this characteristic. In addition, as far as we know, in Brazil, there is little evidence linking sleep duration and TV time with cardiometabolic risk in this population, so we suggest this is one of the first studies that investigate the moderating role of age in the relationship between TV and cardiometabolic risk. The relationship between sleep time and the presence of cardiometabolic risk has also been recently investigated, especially prolonged sleep, highlighting the importance of this investigation.

## Conclusions and implications

In conclusion, TV time and sleep duration are associated with cardiometabolic risk; adolescents with short sleep have higher cardiometabolic risk. In addition, age plays a moderating role in the relationship between TV time and cardiometabolic risk, indicating that in younger adolescents the relationship is stronger compared to older ones. From this, we suggest the need for longitudinal studies that increase the power of inference for a causal relationship, studies that verify the association between long sleep duration and changes in cardiometabolic risk, as well as lifestyle interventions in adolescents to prevent cardiometabolic disorders. Moreover, for future studies, it is suggested to consider the role of other behaviors as potential confounders, such as physical activity and eating habits.

## Data Availability

The database considered in the present study is not publicly available since its information could compromise the subjects’ privacy and consent regarding the research. However, upon request, the corresponding author can provide the data.

## Abbreviations

cMetS: Continuous metabolic syndrome risk score
CRF: Cardiorespiratory fitness test
HDL-c: High-density lipoproteins cholesterol
CI: Confidence interval
SBP: Systolic blood pressure
SPSS: Statistical Package for the Social Sciences
TG: Triglycerides
TV: Television
VO_2peak_: Peak oxygen uptake
WC: Waist circumference
